# A Sex Perspective in Neurodegenerative Diseases: microRNAs as Possible Peripheral Biomarkers

**DOI:** 10.3390/ijms22094423

**Published:** 2021-04-23

**Authors:** Paola Piscopo, Maria Bellenghi, Valeria Manzini, Alessio Crestini, Giada Pontecorvi, Massimo Corbo, Elena Ortona, Alessandra Carè, Annamaria Confaloni

**Affiliations:** 1 Department of Neuroscience, Istituto Superiore di Sanità, Viale Regina Elena 299, 00161 Rome, Italy; valemanzini44@gmail.com (V.M.); alessio.crestini@iss.it (A.C.); annamaria.confaloni@iss.it (A.C.); 2 Center of Gender Specific Medicine, Istituto Superiore di Sanità, Viale Regina Elena 299, 00161 Rome, Italy; maria.bellenghi@iss.it (M.B.); giada.pontecorvi@iss.it (G.P.); elena.ortona@iss.it (E.O.); alessandra.care@iss.it (A.C.); 3 Department of Neurorehabilitation Sciences, Casa Cura Policlinico, Via Dezza 48, 20144 Milano, Italy; m.corbo@ccppdezza.it

**Keywords:** neurodegenerative diseases, biomarkers, microRNAs, sex differences, Alzheimer’s disease, Parkinson’s disease, amyotrophic lateral sclerosis

## Abstract

Sex is a significant variable in the prevalence and incidence of neurological disorders. Sex differences exist in neurodegenerative disorders (NDs), where sex dimorphisms play important roles in the development and progression of Alzheimer’s disease, Parkinson’s disease, and amyotrophic lateral sclerosis. In the last few years, some sex specific biomarkers for the identification of NDs have been described and recent studies have suggested that microRNA (miRNA) could be included among these, as influenced by the hormonal and genetic background. Failing to consider the possible differences between males and females in miRNA evaluation could introduce a sex bias in studies by not considering some of these sex-related biomarkers. In this review, we recapitulate what is known about the sex-specific differences in peripheral miRNA levels in neurodegenerative diseases. Several studies have reported sex-linked disparities, and from the literature analysis miR-206 particularly has been shown to have a sex-specific involvement. Hopefully, in the near future, patient stratification will provide important additional clues in diagnosis, prognosis, and tailoring of the best therapeutic approaches for each patient. Sex-specific biomarkers, such as miRNAs, could represent a useful tool for characterizing subgroups of patients.

## 1. Introduction

Neurodegenerative diseases (NDs) have a huge impact on healthcare systems. They often are multifactorial with a complex pathophysiology, which makes an early diagnosis and identify effected treatments difficult. Several studies suggest that neuronal abnormalities start to appear 10–20 years before the onset of symptoms. Treatments are only symptomatic, and no pharmacological therapies are currently available to stop the underlying disease process [1]. There is a growing need for new tools having the ability to detect patients in their preclinical stage, monitor the disease progression, improve the understanding of various drug mechanisms targeting different pathogenic processes, and identify any response to treatment in a more sensitive and objective way. Using biomarkers, we can detect biochemical changes that appear when neurons begin to die. MicroRNAs (miRNAs) are highly conserved small noncoding RNAs regulating post-transcriptional gene expression. Since their discovery, a large number of miRNAs have been described. They are remarkably stable in human biofluids and easy to manage. As a result, miRNAs have emerged as powerful diagnostic or prognostic biomarkers in the pathophysiology of different neurodegenerative disorders and conditions affecting the central nervous system, in particular in older adults [2,3].

Gender differences have been a focus of interest in a growing number of studies in recent years. Several human conditions show gender or sex differences when considering their pathogenetic mechanisms, their progression, the age of onset, and treatment response. Recent studies suggest that the cause may lay in miRNA expression levels and that these differences might be influenced by a hormonal and genetic background [4]. Ignoring these differences could alter the meaning of the obtained results, as clusters or families of genes or molecular determinants, which have the potential to cause different disease onsets, could be missed or their functions and roles may not be further investigated. Moreover, failing to consider possible differences between males and females in miRNAs and in biomarkers in general could introduce a gender bias in the study.

Therefore, in this review, by analyzing the studies summarized in Table 1 and discussed in Table 2, we have tried to find an answer to the question: “Can we identify sex-specific biomarkers involved in the early and differential diagnosis, and which could, eventually, be useful to treat patients in a personalized way?”.

## 2. microRNAs as Possible Biomarkers in Neurodegenerative Diseases

Biomarkers are powerful tools in biomedical research, and can be useful as supports in selecting the best therapeutic strategies. Testing-specific biomarkers allow monitoring modulations in normal or pathological conditions and give the opportunity to identify diseases in their early stages or to follow them throughout their progression. Clinical symptoms in the early stages might be very subtle, manifesting only when molecular and cellular alterations have already occurred. Thus, there is an increasing effort to develop molecular diagnostic and prognostic markers that meet certain requirements, such as easy accessibility, high specificity and sensitivity, low costs, and applicability to laboratories with standard equipment, for example peripheral biomarkers.

The most well established markers for the detection and monitoring of the preclinical and clinical stages of Alzheimer’s disease (AD) include cerebrospinal fluid (CSF) measures of Aβ42, total tau (t-tau), and phospho-tau (p-tau) [5], while the most promising for the diagnosis and monitoring of the progression of Parkinson’s disease (PD) is probably the assay of alpha-synuclein [6]. Other tools useful to support AD diagnosis are magnetic resonance imaging for hippocampal atrophy [7], the 18F-FDG PET to identify abnormal brain metabolism [8], and amyloid PET to detect the amyloid deposition [9]. However, these tests are invasive and expensive, and thus difficult to adopt in routine clinical practice.

An important novelty in this field is represented by peripheral circulating miRNAs. They are found in peripheral biofluids (saliva, blood, plasma, serum, urine), and therefore are easy to obtain from patients without any substantial risk to health. Moreover, miRNAs are remarkably stable in human biofluids and are easy to manage, thus they are increasingly emerging as promising powerful diagnostic or prognostic biomarkers of different pathophysiological processes [10] in conditions affecting the central nervous system, particularly in older adults. Interestingly, circulating miRNAs and miRNA profiles specific for AD, mild cognitive impairment (MCI) syndrome, PD, and amyotrophic lateral sclerosis (ALS) have been defined and characterized [11,12,13].

MicroRNAs are short non-coding RNAs (20–25 nucleotides), which are emerging as fundamental factors that are mostly displaying their functions at post-transcriptional levels. These short RNA sequences exert their action by binding to the 3’UTR of their target genes to negatively modulate their expression. Depending on the precision of the sequence complementarity, miRNAs can induce translational repression or mRNA degradation.

Each gene transcript might be targeted by several miRNAs and each single miRNA can bind and regulate hundreds of direct targets. Due to their number and complexity, miRNA–mRNA interactions are far from being well demonstrated.

Canonically, miRNAs are encoded as individual or cluster transcripts, with the latter containing several different miRNAs. Many miRNAs are located in the intron region of host protein coding genes [14]. Typically, miRNAs are generated from longer primary miRNA transcripts (pri-miRNA and pre-miRNA), which undergoing two sequential cleavages and passing to the RNase III enzymes Drosha and Dicer, produce the mature and biologically active molecules, which form the silencing complex together with the argonaute (AGO) protein.

It is important to note that miRNAs have unique characteristics, which make them possible candidate biomarkers. Differing from coding genes, miRNAs do not require translation and their expression level usually corresponds to their activity. Moreover, they are highly stable and easily preserved in biological specimens, thus making their analysis more informative than the analysis of longer RNAs. Even more importantly, growing data indicate that circulating miRNAs are representative of the releasing tissues and their detection in plasma or serum supports their use in non-invasive diagnostic or prognostic methods, which can eventually lead to the identification of therapeutic targets.

## 3. MicroRNAs in the Brain: From Function to Gender Differences

Besides their relevant physiological roles, the deregulation of miRNAs is involved in the onset and progression of several human pathological conditions, including neurodegenerative diseases. Significant sex differences have been associated with physiological and pathological miRNA functions. Differences in their expression levels can lead to a propensity to develop specific diseases, such as the higher risk of developing Alzheimer’s disease observed in females.

Males and females have considerable differences in their hormonal and genetic profiles [4], and these variables are known to impact on microRNAs. In particular, sex steroid hormones, such as estrogens, progesterone, and testosterone, are known to regulate the expression of several microRNAs [15,16,17,18].

In recent years, a growing number of studies have highlighted the impacts of sex-related microRNA expression on brain development [19], with sex hormones showing the ability to bind to nuclear hormone receptors in order to induce a direct or indirect alteration in gene expression [20]. Furthermore, during miRNA biogenesis, sex hormones could also have a huge impact on miRNAs regulation. In fact, estradiol has been proven to alter the expression of AGO, Drosha, and Dicer [21].

Surprisingly, despite genomic distribution analysis showing that the human X chromosome has a higher density of miRNAs compared to autosomes and the Y-chromosome [22,23], no literature data are currently available on sex-chromosome-related microRNAs implicated in NDs.

Due to the extreme complexity of the miRNA-based regulatory circuitries, further in depth analyses will be required in order to understand in a more definite way their physiological and pathological activity, and to confirm their promising roles as biomarkers.

Focusing on the hot spot of this review, very high-throughput sequencing experiments were interpreted, suggesting that the number of miRNAs expressed in the human brain is over 1000, although currently only ~550 have been annotated in all humans [24]. Certain miRNAs and other non-coding RNAs have been suggested to act as “complexity multipliers” to help translate the ~25,000 human protein-coding genes into the human cerebral cortex, thus participating in neurodevelopmental and neurophysiological network integration [25]. The expression of brain miRNA changes continuously during brain development, with some miRNAs being enriched during the early phase of brain maturation and others during the late phase. Many miRNAs are highly expressed in the adult nervous system in a spatially and temporally controlled way in both normal physiology and in certain pathological conditions. Indeed, miRNAs have been implicated in various aspects of dendrite remodeling and synaptic plasticity, as well as in experience-dependent adaptive changes of neural circuits in postnatal developmental and the adult brain [26].

Moreover, some miRNAs are “exclusive” to different cell populations. As an example, in the human hippocampus, miR-124 and miR-320 are expressed mainly by neurons, in the substantia nigra miR-320 is mainly expressed by pigmented cells, while miR-107 is barely expressed by the same cell types [27]. The functions of brain miRNAs are clearly not related only to cell fate determination along developmental lineages, but also to neuroplasticity and to many other neurobiological functions, including the regulation of the synthesis of synaptic proteins, the morphogenesis of the dendritic spine, and in plasticity-related diseases [28].

Ziats and colleagues observed significant sex differences in the expression of 40 miRNAs in the prefrontal cortex of males and females. The majority of sex-biased gene expression occurred in adolescence (65%), suggesting that miRNA-targeted gene expression differences become most pronounced around puberty, probably due to the hormonal influence of this life stage [29]. Importantly, Murphy and colleagues observed a differential expression of microRNAs according to sex in the development of the cortex in rats [19]. They performed expression studies in male and female cortices isolated from postnatal day 0 (P0), postnatal day 7 (P7), and adult rats, showing differential miRNA levels between males and females at each developmental stage. They focused in particular on the miR-200 family, which has a “biphasic” expression, being higher in female rats at P0, inverting its expression at P7, and being higher in male rats during adult life. Moreover, Cui and colleagues identified 73 female-biased miRNAs and 163 male-biased miRNAs in several tissues, including brain tissue [30].

## 4. Sex Differences in Neurodegenerative Diseases

Human pathological conditions that show differential incidence rates, clinical manifestations, and progression according to sex can be extremely interesting as a focus of research [31]. In particular, sex is a significant variable in the prevalence and incidence of neurological disorders [32]. Sex differences can be observed in age at onset, disease severity, progression, and response to treatment [33]. The biological factors underlying these sex disparities are currently poorly understood. Although traditionally attributed to hormonal effects, recent evidence has described the role of genetics, and in particular of the sex chromosomes in these sexual dimorphisms.

The response of the central nervous system (CNS) to sex steroids is complex and depends on the type, amount, and cellular distribution of sex hormone receptors. Female sex hormones, and in particular estrogens, have neuroprotective effects on the CNS [34,35], while more controversial data have been reported on the effect of male hormones [36,37].

Sex differences can be also observed in neurodegenerative disorders, where sex dimorphisms play an important role in the development and progression of AD, PD, and ALS. Considering sex as a biological variable, research promises to advance our understanding of the pathophysiology and treatment of these conditions.

AD is a multifactorial, degenerative condition characterized by progressive cognitive decline. It is one of the most common neurodegenerative disorders, affecting more than 45 million individuals throughout the world. The disease is defined and diagnosed based on the presence of amyloid-β (Aβ) plaques and neurofibrillary tangles (NFTs), which lead to oxidative stress, neuroinflammation, neural dysfunction, and neurodegeneration. Interestingly, Aβ peptides and tau protein clearance could also have main roles in the development of AD and others neurodegenerative pathologies [38,39,40,41]. Women and men have been reported to have differential vulnerability, pathogenesis, and clinical presentation. The prevalence of AD has been estimated to be higher in women than in men (2:1 female/male ratio) [42], with postmenopausal women contributing to over 60% of all affected subjects [43]. Despite a number of contrasting reports on sex-related differences in the incidence rates of AD [44,45,46,47,48,49], overall the evidence seems to homogeneously point to a higher incidence of AD in older women [50,51]. However, the biological causes underlying the sex-specific phenotypes in AD and the other neurodegenerative diseases are not yet well understood.

Some of the evidence has demonstrated that non-nuclear membrane estrogen receptor α (mERα) can play a role in the mechanism; mERα regulates a coordinated framework of defense strategies aimed at counteracting chronic oxidative imbalance, mitochondrial dysfunction, and brain energy dysmetabolism, which is a causative factor of neuronal cell degeneration, as well as in determining neuron survival [52,53]. Estrogens have a wide range of beneficial actions in the brain and in other tissues, and their loss may increase the risk of AD. Many studies suggest that reductions of estrogen in adulthood are associated with an increased risk of AD in women [54].

As for male sex hormones, similarly to the association between low estrogen levels and an increased risk of AD in women, lower testosterone levels may lead to a higher risk of AD in men. Moffat and colleagues [55] showed that testosterone depletion is an early component of the risk of AD in men, at least ten years prior to the clinical diagnosis of dementia. Importantly, the loss of testosterone in brain tissue is a key component of the disease process [56,57], and this mechanism is sex-specific because testosterone levels are not significantly different in women with AD [57].

PD is a progressive neurodegenerative disorder. Its main pathological feature is the progressive loss of midbrain dopaminergic (DA) neurons in the substantia nigra pars compacta (SNc) and the presence of misfolded a-synuclein-positive cytoplasmic inclusions, called Lewy bodies, in the surviving neurons [58,59].

PD has a 1.5 higher frequency in men than women [60,61] at all ages and for all ethnicities [62], but it has a higher mortality rate and faster progression in women [63].

Several studies have reported certain specific sex differences in the clinical and cognitive characteristics of PD. PD in women starts with a more benign phenotype, probably due to the protective effect of estrogens, as the age of onset is correlated with the fertile life span. Moreover, gonadal hormones and sex chromosomes might modulate the risk of developing PD by influencing epigenetic mechanisms [62,64,65]. Preclinical evidence [62,66,67] suggested a potential neuroprotective effect of estrogens on dopaminergic neurons and in preventing brain damage through anti-inflammatory, anti-oxidative, and anti-apoptotic mechanisms [68,69], along with possible inhibitory effects on the formation and stabilization of α-synuclein fibrils, a key pathological feature of PD. However, the progression of the disease in women leads to a higher risk of developing highly disabling treatment-related complications, such as motor and non-motor fluctuations, as well as dyskinesia [66,70].

As for AD, higher levels of estrogens are inversely correlated with the severity of PD symptoms, and women who experienced early natural or surgical menopause show a higher risk of PD [71].

Conflicting results have been observed regarding the functional role of male sex hormones in PD. In particular, one study reported that the prevalence of testosterone deficiency in male PD patients is higher than in men during their healthy aging [72]. Whether this imbalance is a precondition to or a consequence of PD is currently unknown.

Well-established effects of sex have been also reported on certain pathological and epidemiological features of ALS. ALS is a complex neurodegenerative disease primarily characterized by the progressive loss of both upper and lower motor neurons in the brain and spinal cord, leading to fatal paralysis and respiratory failure [73]. As for PD, males are more susceptible to the disease [74] and sometimes develop more severe symptoms [75]. Both the incidence and prevalence rates of ALS in males and females depend on the age classes. However, older patients with ALS are more likely to be female, suggesting that menopause and associated hormonal changes could be involved in the higher frequency of the disease in older women [76].

Effects of sex on the most common clinical forms of the disease and types of symptom onset have been observed. A higher proportion of spinal onset was reported amongst men, while a higher frequency of bulbar onset was reported amongst women [77].

Several studies have described a protective effect of hormones in ALS murine models [77]. In fact, female hormones prevent cell death and reduce the neuroinflammation in neural and muscular cells. In particular, a study performed on *SOD1* G93A (the most relevant human mutation in ALS pathogenesis) in mice showed that progesterone slowed down the progression of the disease and extended the life span of male mice with ALS, due to the activation of autophagic degradation of mutant SOD1 [77]. As for the effects of male sex hormones on the risk of ALS, testosterone efflux seems to have a role during prenatal life, irrespective of sex, in the lifelong risk of ALS, with increased prenatal levels of testosterone being a potential independent risk factor [78]. Importantly, ALS patients who had higher levels of testosterone and a lower progesterone/free testosterone ratio showed more rapid deterioration of respiratory parameters [79].

However, sex differences in the clinical features of ALS should be further investigated to understand the molecular mechanisms at the base of such dimorphisms and to develop more effective treatment options for ALS.

## 5. Peripheral Sex-Related miRNAs and Neurodegenerative Diseases

In the last few years, certain sex-specific biomarkers for the identification of NDs have been described [11], such as progranulin (PGRN) levels as markers in the pathogenesis of AD [80]. Among them, certain miRNAs have been identified as sex-related biomarkers, which are influenced by the hormonal and genetic background [4]. Table 1 and Table 2 summarize certain characteristics of studies reporting sex-related miRNA differences in peripheral samples of subjects with AD, PD, and ALS.

### 5.1. Alzheimer’s Disease and Frontotemporal Dementia

We found several studies investigating some miRNAs as peripheral sex-specific biomarkers for AD and frontotemporal dementia (FTD).

Grasso and coauthors when analyzing plasma samples in a population of 48 patients with FTD and 46 healthy controls found significant downregulation of miR-663a, miR-502-3p, and miR-206 (*p* = 0.0001, *p* = 0.0002, *p* = 0.02, respectively) in FTD patients. However, stratification of data according to sex showed significant differences in miR-663a and miR-502-3p in both males and females and significant differences in miR-206 only in male subjects. To obtain a discriminating measure between FTD subjects and healthy controls (HCs), they calculated a combined score of three miRNAs when applying a Bayesian approach and obtained a classifier accuracy of 84.4%. Combined miRNA levels showed higher sensitivity (100%) and specificity (87.5%) in distinguishing FTD patients from HCs in males versus females. Interestingly, miR-502-3p is located on the X-chromosome (miRbase) [81]. Moreover, they found a significant difference in let-7e-5p levels between HCs and FTD females, but no difference was found between males and in the overall population.

Sex-specific differences in miR-206 were also evaluated in a longitudinal study including a five-year follow up, with the aim of identifying a biomarker capable of predicting the conversion from MCI to dementia by analyzing miRNA expression in 96 plasma samples (30 MCI patients, 35 AD patients, 31 HCs). The results showed miR-206 as the most promising marker, however with no sex-related differences [82].

Another study on plasma samples investigated miR-127-3p levels in patients with FTD (54 samples) compared with patients with AD patients (20 samples) and controls (53 samples). The results showed an underexpression of this miRNA in FTD samples. After stratifying data according to sex, the same trend was observed in both males and females, with good ROC diagnostic values [83]. The area under the curve (AUC) values of miR-127-3p for discrimination between FTD and HC was 0.7684 in males and 0.8264 in females, and for discrimination between FTD and AD was 0.9263 in males and 0.8714 in females.

Denk and colleagues [84] investigated the differential expression of miRNAs in serum samples of 38 HCs, 48 patients with FTD, and 47 patients with AD. Of the investigated miRNAs, miR-103a-3p, miR-106a-5p, and miR-1246 were the best at discriminating between FTD and AD in males but not in females. Similar results were found in CSF samples.

Interestingly, certain sex-specific differences in diagnostic accuracy were reported in the study by Siedlecki-Wullich and colleagues [85]. They replicated an analysis of biomarkers in plasma samples of subjects with AD, FTD, and MCI. The aim of the study was to validate certain miRNAs as biomarkers capable of discriminating between the three conditions. The results showed a significant upregulation of miR-92a-1-3p in AD compared with control, overexpression of miR-181c-5p and miR-210-3p in both AD and MCI, while no miRNA was dysregulated in FTD. The ROC diagnostic value for males was better than for female, in particular the specificity was higher in males, while sensitivity was higher in females. The AUC values for all three miRNAs showed that their ability to discriminate between MCI and controls switched from a value of 0.89 with 84.62% sensibility and 85.71% specificity in females to a value 0.96 with 100% sensibility and 85.71% specificity in males. The AUC values for the three miRNAs in discriminating between AD and HC increased from 0.85 (92.86% sensibility, 71.43% specificity) in females to 0.94 (75% sensibility, 100% specificity) in males [85].

However, not all studies on sex differences in miRNA levels reported positive results. Galimberti and colleagues performed a study in 2014 on serum samples from 22 patients with AD, 10 with FTD, 18 with non-inflammatory conditions, and 8 controls with inflammatory diseases. They found some downregulated miRNAs in AD versus all other groups, but no sex-related differences were reported [86]. Another study was performed on blood samples from a large population of 465 subjects, including two control groups (healthy and other neurological diseases such as Parkinson’s disease, schizophrenia, or bipolar disorders) compared with patients with AD and MCI. The results showed a dysregulation of almost twelve miRNAs in patients with Alzheimer’s disease but none with differential expression in males and females [87]. Similarly, Cosìn-Tomàs et al. found two downregulated miRNAs in plasma samples from patients with AD compared to healthy controls, describing no sex-related differences [88]

### 5.2. Parkinson’s Disease

Looking at PD, a study performed on 667 plasma samples obtained from 269 patients with PD, 222 HCs, and 176 neurodegenerative disease controls (NDC) [89] reported a higher expression of miR-132 in neurons and altered levels in other neurodegenerative diseases. The results showed a significantly higher expression of this miRNA in males with PD but not in females with PD compared to controls, while no differences were observed between PD and ND controls. Further analyses of miR-132 and Nurr1 levels (a protein involved in dopamine regulation) showed a negative correlation between higher levels of miR-132 and lower levels of this protein, suggesting a possible role of this miRNA in Nurr1 regulation.

The MiR-132 family, along with miR-134, was reported in a validation study, where 50 plasma samples collected from MCI patients and 50 from healthy controls were analyzed. The discrimination between controls and MCI according to ROC was more accurate in males for miR-132 family members (miR-128, miR-132, miR-87) and in females for miR-134 family (miR-134, miR-323, miR-382) [90].

A study on serum samples [91] reported the analysis of the miRNA-29 family in a population of 80 PD patients and 80 HCs. The levels of miR-29a and miR-29c were significantly higher in females than in males in both controls and in patients with PD.

In another study on salivary samples, Chen and colleagues [92] enrolled 30 patients with PD and 30 HCs and analyzed microRNAs that regulate the deglycase DJ-1 gene expression, finding no differences between sexes.

### 5.3. Amyotrophic Lateral Sclerosis

Regarding ALS, Toivonen [93] analyzed the serum samples of 12 patients with ALS and 12 HCs. When comparing results from male patients with male controls, no significant differences were observed, although miR-206 was close to significance (3.4-fold upregulation, *p* = 0.1). When comparing results between female patients and controls, miR-206, miR-133b, and miR-145 were upregulated (5.4-fold *p* = 0.02; 2-fold *p* = 0.03; 1.4-fold *p* = 0.04). As described above, sex-related miR-206 levels were investigated in two other studies on FTD [81] and AD [82].

## 6. Conclusions and Futures Perspectives

Several drugs for the treatment of NDs have been investigated in clinical trials in the last 15 years, but all with inconclusive results. No new medications have been recently approved by the drug regulatory agencies, and effective pharmacological treatments for NDs are still lacking. A possible reason for this failure might be linked to the idea that only very early interventions can be effective in these conditions. However, another possible reason could be the view of neurodegeneration as a homogeneous group of diseases. It is important to underline that there is no “one-size-fits-all” treatment that can be useful for all patients; rather, according to the concept of precision medicine, treatments must be personalized, addressing the etiology of the disease in each person. In this context, it could be useful to characterize specific subpopulations of patients by considering genetic and epigenetic factors, and gender differences could be one of these factors. Epidemiological data confirm strong male–female disparities in the onset and progression of AD, PD, and ALS. Although these differences could be partly due to gender-related specific behaviors, recent results also indicate biological variations as key players. Among them, microRNAs seem to have an important function in defining a molecular sex-related asset due to their sex hormone regulation. In this review, we reported sex-specific differences of peripheral miRNA levels in some NDs. As an example, miR-206, which is underexpressed in males with FTD and overexpressed in males with ALS, is regulated by hormones [94,95,96]. MiR-132 was found to be a particularly expressed miRNA in the cerebellum of males [97], which might explain why Yang and co-authors found it to be more upregulated in plasma samples of male patients with PD. Likewise, miR-29a and miR-29c were found to be more expressed in the cerebellum of females, which might link to the upregulation in the sera of female PD patients [91].

However, few papers have shown significant differences between miRNA expression levels in males and females in NDs. This may not necessarily be due to a lack of sex-specific correlation, but to the fact that most of these studies did have the primary objective of investigating gender differences, meaning sex was not considered as part of the investigated biological factors. Moreover, the number of studies that have performed a subgroup analysis of males and females is very low compared to the large number of papers published on peripheral miRNAs. Failing to consider the possible differences between males and females in the evaluation of biomarkers such as miRNAs could introduce a sex bias in studies, increasing the risk of missing some of these sex-related specificities. To date, no miRNA has been practically implied in neurodegenerative diseases.

This could be due to the complexity of these pathologies, but also to the variability of the types of study, the diagnostic criteria used for enrolment, the sizes of the populations studied, the types of tissue chosen, and the methodologies used to study the biomarkers. Future studies should aim to adopt a more thorough methodology to consider sex among biological variables when studies on biomarkers are designed.

## Figures and Tables

**Table 1:** Main features of studies included in the review.

Studies	Country	Specimen	Participants	Sample Size		Age (Mean ± SD)		Sex (M/F)	
				Cases	Controls	Cases	Controls	Cases	Controls
Alzheimer’s Disease and Frontotemporal Dementia									
[81]	Italy	Plasma	FTD/HC	48	46	72 ± 8	73 ± 7	20 (42%)/28 (58%)	18 (39%)/28 (61%)
[87]	USA, Germany	Blood	AD MCI HC/OND	145 AD 38 MCI	214 HC 68 OND	72.9 ± 9.8 71.6 ± 6.2	68.9 ± 7.8 57 ± 18.7	76 (52%)/69 (47%) AD 18 (47%)/20 (53%)MCI	103 (48%)/111 (52%) HC 23 (34%)/45 (66%) OND
[84]	Germany	CSF, Serum	FTD/AD/HC	96 FTD 95 AD	82	65 ± 9.2 65 ± 9.3	64 ± 11.3	78 (81%)/18 (19%) FTD 69 (73%)/26 (27%) AD	58 (71%)/24 (29%)
[83]	Italy	Plasma	FTD/AD/HC	54 FTD 20 AD	53	70.3 ± 8.8 71.3 ± 8	71.6 ± 7.3	19 (35%)/35 (65%) FTD 10 (50%)/10 (50%) AD	20 (38%)/33 (62%)
[88]	Spain	Plasma	AD/PAD HC	36 AD 36 PAD	36	68.1 68.9	66.64	15 (42%)/21 (58%) AD 18 (50%)/18 (50%) PAD	16 (44%)/20 (56%)
[86]	Italy	Serum CSF	AD/FTD NINDC/INDC	22 AD 10 FTD	18NINDC 8 INDC	72.7 ± 0.6 69.4 ± 0.6	68.8± 0.71 66.5± 0.22	8 (36%)/14 (64%) AD 2 (20%)/8 (80%) FTD	13 (72%)/5 (28%) NINDC 0 (0%)/8 (100%) INDC
**Mild Cognitive Impairment (MCI)**									
[82]	Spain	Plasma	MCI/AD/HC	30 MCI 35 AD	31	76.8 ± 4.0 84.6 ± 3.5	75 ± 4.7	13 (43%)/17 (57%) MCI 14 (40%)/21 (60%) AD	17 (55%)/14 (45%)
[85]	Spain	Plasma	MCI/AD/HC	26 MCI 56 AD	14	72 ± 8.5 77.8 ±6.7	68.3± 8.99	10 (38%)/16 (62%) MCI 15 (27%)/41 (73%) AD	7 (50%)/7 (50%)
[90]	USA	Plasma	MCI/HC	50	50	68.2	65.1	21 (42%)/29 (58%)	26 (52%)/24 (48%)
**Parkinson’s disease**									
[92]	China	Saliva	PD/HC	30	30	63.2 ± 10.2	59.7 ± 12.8	20 (67%)/10 (33%)	16 (53%)/14 (47%)
[89]	China	Plasma	PD/HC/NDC	269	222 HC 176 NDC	66.1 ± 0.61	66.2 ± 0.61 66.2 ± 0.74	157 (58%)/112 (42%)	130 (59%)/92 (41%) HC 105 (60%)/71 (40%) NDC
[91]	China	Serum	PD/HC	80	80	64.0 ± 5.8	63.3 ± 5.4	48 (60%)/32 (40%)	48 (60%)/32 (40%)
**Amotrophic Lateral Sclerosis**									
[93]	NR	Serum	ALS/HC	12	12	57.0 ± 12.2	54.0 ± 14.5	6 (50%)/6 (50%)	6 (50%)/6 (50%)

AD: Alzheimer’s Disease; MCI: Mild Cognitive Impairment; FTD: Frontotemporal Dementia; HC: Healthy Controls; PD: Parkinson’s Disease; NDC: Neurodegenerative Disease Controls; OND: Other Neurological Diseases; PAD: Preclinical AD; NINDC: Non-Inflammatory Disease Controls; INDC: Inflammatory Disease Controls; ALS: Amyotrophic Lateral Sclerosis.

**Table 2:** Summary of differences between sexes in miRNA levels from studies including patients with NDs.

Studies	Specimen	miRNA	Differences between Sexes	Possible Roles
Alzheimer’s Disease and Frontotemporal Dementia				
[81]	Plasma	miR-206 miR-502-3p, miR-663a let-7e-5p	Downregulated in FTD vs. HC males (*p* < 0.05). Underexpressed both in males (*p* < 0.01; *p* < 0.1) and females (*p* < 0.1; *p* < 0.01). Downregulated in females with FTD (*p* < 0.05)	miR-206: synaptogenesis and neurogenesis. miR-502-3p: schizophrenia. miR-663a: inflammatory response, neuronal differentiation and development
[87]	Blood	10 miRNAs differentially expressed	No differences	Notch signaling pathway.
[84]	CSF, Serum	41 miRNAs differentially expressed: miR-103a-3p, miR-106a-5p, miR-1246	No differences in expression level. AUC values better in males than females (miR-103a-3p: AUC = 0.80; miR-106a-5p: AUC = 0.80; miR-1246: AUC = 0.85)	miR-103a-3p and miR-106a-5p: involved in neuropathological processes by targeting LRP1, CDK5R1, DLG4 (Chang et al., 2017), APP, BACE1, PSEN1 (Yilmaz et al., 2016). miR1246: not yet studied
[83]	Plasma	miR-127-3p	Downregulated in FTD with respect to AD or HC both in males (*p* < 0.0001; *p* < 0.05) and females (*p* < 0.001; *p* < 0.0001)	neuronal proliferation, differentiation and development
[88]	Plasma	miR-34a-5p, miR-545-3p	No differences	miR-34a-5p: synaptic plasticity, glutamate receptors, potassium/sodium channels, antiapoptotic protein BCL-2 and SIRT1, inhibits pentose phosphate pathway in neurons and the mitochondrial functions. miR-545-3p targets the mRNA of the APOE receptor involved in Aβ clearance
[86]	Serum, CSF	miR-125b, miR-6b	No differences	cell-cell communication
**Mild Cognitive Impairment**				
[82]	Plasma	miR-206	No differences	neuronal survival, differentiation and signals transmission
[85]	Plasma	miR-92a-1-3p, miR-181c-5p, miR-210-3p	No differences in expression levels. The ROCs show better diagnostic power in males (AUC = 0.957) than in females (AUC = 0.821)	synaptic plasticity, especially the glutammaergic ones
[90]	Plasma	6 miRNAs analysed: miR-132 sets miR-134 sets	No differences in expression levels. Better accuracy for male miR-132 sets of ROC (AUC = 0.99) compared with female (AUC = 0.98) and general ROCs (AUC better value = 0.97), and for female miR-134 sets of ROC (AUC = 0.96) compared with male (AUC = 0.90) and general ROCs (AUC better value = 0.93)	miR-132 and miR-134 family: Tau network and neuron growth
**Parkinson’s disease**				
[92]	Saliva	miR-874	No differences	Synaptic function, morphogenesis, neurotransmitter regulation
[89]	Plasma	miR-132	Upregulated in male PD (*p* < 0.01).	Negative correlation with Nurr 1
[91]	Serum	miR-29a, miR-29b, miR-29c	Upregulated in female PD (*p* = 0.041; *p* = 0.0062; *p* = 0.0107).	Neurons survival, maturation, proliferation, synaptic plasticity, and morphogenesis
**Amotrophic Lateral Sclerosis**				
[93]	Serum	miR-206	Upregulated in female ALS (*p* = 0.02)	synaptogenesis and neurogenesis

AD: Alzheimer’s Disease; MCI: Mild Cognitive Impairment; FTD: Frontotemporal Dementia; PD: Parkinson’s Disease; ALS: Amyotrophic Lateral Sclerosis; AUC: Area Under the Curve.

## Data Availability

Not applicable.
